# The effect of surgical cure of primary hyperparathyroidism on cardiac electrical activity: a cross−sectional study

**DOI:** 10.3389/fendo.2025.1601897

**Published:** 2025-07-23

**Authors:** Piotr Kmieć, Izabela Karwacka-Bujak, Michał Bohdan, Renata Świątkowska-Stodulska, Krzysztof Sworczak

**Affiliations:** ^1^ Department of Endocrinology and Internal Medicine, Medical University of Gdańsk, Gdańsk, Poland; ^2^ Department of Endocrinology and Internal Medicine, University Clinical Center, Gdańsk, Poland; ^3^ 1st Department of Cardiology, Medical University of Gdańsk, Gdańsk, Poland

**Keywords:** primary hyperparathyroidism, parathyroidectomy, hypertension, premature cardiac complexes, electrocardiography

## Abstract

Cardiovascular complications are not assessed routinely in the management of primary hyperparathyroidism (pHPT), nor do they constitute indications for surgical treatment of this disorder. Research concerning the effects on cardiac electrical activity in PHPT is scarce. In the current study, 45 consecutive pHPT patients with hypercalcemia and elevated parathyroid hormone levels were assessed clinically, biochemically and by 24-h ECG monitoring before, one and six months after curative parathyroidectomy (PTX). There were 41 female and four male subjects, their mean age was 54.6 ± 14.6 years. 20 patients were normotensive and 25 had previously or newly diagnosed hypertension. Patients without hypertension compared to the hypertensive ones had lower BMI: 23.2 (20.3-25.4) versus 26.7 (24.8-28.4), higher total calcium: 11.9 ± 0.8 versus 11.3 ± 0.9 mg/dL, and shorter QTc: 418 ± 17 versus 436 ± 17 ms, p<0.001. Before surgery, Ca and PTH correlated negatively with QTc. Upon curative PTX, the median number of supraventricular premature beats (SVPBs) and ventricular premature beats (VPBs) decreased significantly, which was paralleled by a 37% decrease in the prevalence of clinically significant SVPBs (>76 per 24h), and a 29% decrease in the number of patients with more than 3 VPBs/24h six months after surgery. QTc increased from 428 ± 19 before to 441 ± 17 ms after PTX. The change in the median number of SVPBs and VPBs was comparable between patients with versus without HT. Curative PTX normalizes QTc, reduces supraventricular and ventricular extrasystoles in patients with hypercalcemic pHPT.

## Introduction

In the course of primary hyperparathyroidism (pHPT) chronic excess parathyroid hormone (PTH) secretion results in abnormal calcium-phosphate homeostasis and bone dysmetabolism. Currently, in developed countries, pHPT is mostly (85%) diagnosed in asymptomatic patients, and classic complications of the disorder such as bone lesions, osteoporosis and nephrolithiasis occur rarely (1, 2). While cardiovascular (CV) disorders may constitute the primary complications in the course of pHPT, prospective studies are needed to determine if they should be considered indications for parathyroidectomy (PTX) (3).

pHPT has been associated with increased CV morbidity and mortality compared to the general population (4). Patients with this endocrinopathy have a higher prevalence of hypertension (HT), develop left-ventricular (LV) hypertrophy, valvular heart disease, and conduction abnormalities (5–7). Research concerning the prevalence of arrhythmias in pHPT patients is scarce: neither standard ECG nor Holter ECG monitoring are routinely performed in the management of this endocrinopathy. Still, hypercalcemia does affect the repolarization of cardiomyocytes, which manifests by shorter ST segment and QT time, while increased occurrence of ventricular premature beats (VPBs) may result in major arrhythmia (5).

We recently reported PTX was associated with improvement of LV function and HT control in 45 patients with pHPT (8). Here, we present results of a second part of the study, which aimed at assessing cardiac electrical activity by 24-h Holter ECG in pHPT patients before and after successful PTX.

## Subjects and methods

### Study participants and protocol

The Independent Bioethics Committee for Scientific Research of the Medical University of Gdańsk approved this study on July 2, 2015 (approval no NKBBN/278/2015). Every participant was informed extensively on the protocol and agreed in writing to participate in the study.

In this single-center, cross-sectional study, subjects were recruited in an endocrine outpatient clinic of a tertiary hospital among adults referred due to hyperparathyroidism between 2015 and 2020. Inclusion criteria were hypercalcemia above 10 mg/dl and serum PTH above 69 pg/ml, i.e., respective upper reference range limits applied by the Central Clinical Laboratory of the University Clinical Center of the Medical University of Gdańsk. The following exclusion criteria were adopted: secondary and tertiary hyperparathyroidism, active malignancy, use of a hypocalcemic drug, albuminemia below 35 g/L (lower limit of normal), significant CV disease other than primary HT (in particular atherosclerotic CV disease, heart failure, symptomatic valvular disease or arrhythmia), estimated glomerular filtration rate (eGFR) below 50 mL/min./1.73 m^2^, poor physical condition, and lack of indication or patient’s consent for PTX.

In all enrolled patients, an enlarged parathyroid gland was identified by ultrasound and/or scintigraphy. Histopathological examination revealed a single adenoma in 63% and single-gland hyperplasia in 37% of cases. The treatment was assessed as effective based on normalization or at least 50% reduction of serum PTH and normocalcemia after parathyroidectomy. Patients underwent clinical (interview, physical examination), laboratory, standard ECG, 24-h ECG registration assessment at three timepoints: 1) before, 2) one, and 3) six months post-PTX (along with 24-h ambulatory BP measurements and TTE as reported previously) (8).

### Laboratory examinations

Morning (8–10 a.m.) blood samples were obtained by venipuncture from an upper extremity, centrifuged, and serum/plasma were frozen at -20 degrees C prior to laboratory examinations. Standard methods were used to determine PTH, Ca and phosphate (P) (among others) on a Siemens Immulite 1000 Immunoassay System and an Abbott Architect analyzer (spectrophotometric method). Normal ranges were adopted from manufacturer’s recommendations.

### 24-hour ECG monitoring

ECG monitoring was performed with the Philips Digi Trak XT device with signals from five leads. Patients were requested to record the time of onset and duration of symptoms such as palpitations, dizziness, syncope and irregular heart rate (HR). Standard parameters were analyzed: circadian HR, number of supraventricular premature beats (SVPBs) and ventricular premature beats (VPBs), occurrence of ventricular and supraventricular arrhythmias, number of pauses longer than two seconds, duration of the QRS complexes, corrected QT time (QTc), and ST segments along with the assessment of their morphology. Bradycardia was defined as a decrease in the HR below 50 beats per minute (bpm), and tachycardia as a HR of at least 100 bpm [80]. Horizontal or down-slopping ST segment depression of ≥1 mm for ≥1 minute was considered significant [80]. The number of SVPBs considered as clinically significant was above 76 per 24 hours, since it was associated with mortality in the general population (9). During Holter ECG no patient complained of dizziness, syncope, palpitations or chest pain. Prior to enrollment, bradycardia and first-degree atrioventricular (AV) block had been diagnosed in one patient.

### Statistical analysis

Data were analyzed using GraphPad Prism version 10, with one exception (Cochran’s q test). Statistical tests were selected based on data distribution. Normality was verified using the Kolmogorov-Smirnov test. For data following a Gaussian distribution, Student’s t-test was used for comparisons between two groups, and repeated measures ANOVA with *post-hoc* Tukey’s test was used for paired data from three examination timepoints. Data with non-normal distributions were tested using the Mann-Whitney U and Friedman’s (with *post hoc* Dunn’s multiple comparison) tests. Pearson’s and Spearman’s methods were used to test correlations depending on distribution; their significance was verified with a dedicated test. For binary data, Fisher’s exact test and Cochran’s q test with McNemar’s *post-hoc* test for multiple comparisons (three examination timepoints) with Holm’s correction were applied using RStudio 2024.12.1 Build 563 software. Changes in proportions between patients without and with HT before and after surgery were tested with the z-test for two independent proportions. Results were reported as number (percentage, %), mean ± standard deviation (SD) or median (interquartile range, IQR).

In one patient, Ca concentration of 10.9 mg/dL six months post-PTX was fixed at 10 mg/dL (upper range of normal), since hypercalcemia resulted from iatrogenic calcium carbonate and alfacalcidol therapy for transient post-surgical hypoparathyroidism.

## Results

In the study period, 85 potentially eligible patients were referred. pHPT was excluded in 21 patients, seven were lost to follow-up seven were excluded due to biphosphate therapy, five were not eligible for surgery. Therefore, 45 patients were included in the study. **Table 1** contains clinical, laboratory and Holter ECG data. The cohort was predominantly female: 41 out of 45 (91%). Mean age was 54.6 ± 14.6 years. Almost half of study participants (22) had normal bone mineral density, osteopenia was present in 8, and osteoporosis in 15 patients. HT was present in 25 subjects, while 15 were active smokers.

**Table 1:** Demographic (baseline), laboratory and Holter ECG parameters before and after PTX.

Parameter	All patients				Normotensive patients				p^$^	Hypertensive patients			
n	45				20				–	25			
Age [years]	54.6 ± 14.6				51.1 ± 16				n.s.	57.4 ± 13.1			
BMI	25.2 (22.1-27.3)				23.2 (20.3-25.4)				0.001	26.7 (24.8-28.4)			
M:F ratio	4:41				2:18				1	2:23			
	pre-, one and six months post-PTX			p	pre-, one and six months post-PTX			p	–	pre-, one and six months post-PTX			p
Ca [mg/dL]	11.5 ± 0.9	9.6 ± 0.6 *	9.5 ± 0.4 *	<0.001	11.9 ± 0.8	9.6 ± 0.6	9.4 ± 0.4	<0.001	0.03	11.3 ± 0.9	9.6 ± 0.5	9.6 ± 0.4	<0.001
P [mg/dL]	2.2 ± 0.2	3 ± 0.6 *	3.1 ± 0.6 *	<0.001	2.2 ± 0.2	3 ± 0.4	3.1 ± 0.5	<0.001	n.s.	2.2 ± 0.8	3 ± 0.7	3.2 ± 0.7	<0.001
PTH [pg/mL]	144 (91)	49 (41)^***^	35 (27)^***^	<0.001	192 ± 129	42 ± 22^***^	36 ± 17^***^	<0.001	n.s.	141 ± 62	60 ± 42^***^	42 ± 23^***^	<0.001
HR [bpm]	79.8 ± 9.6	75.1 ± 7.8^**^	75.1 ± 7.4^**^	<0.001	81.8 ± 8.8	76.2 ± 4.3^***^	76 ± 5.4^**^	<0.001	n.s.	81.4 ± 9.9	76.4 ± 7.76	75.7 ± 7.4	n.s.
QRS [ms]	94 (88-99)	94 (87-99)	94 (89-98)	n.s.	95 (90-98)	94 (87-98)	93 (89-98)	n.s.	n.s.	93 (86-99)	92 (87-99)	94 (88-98)	n.s.
QTc [ms]	428 ± 19	441 ± 17^***^	441 ± 17^***^	<0.001	418 ± 17	439 ± 18^***^	440 ± 17^***^	<0.001	<0.001	436 ± 17	442 ± 17	443 ± 17	n.s.
SVPBs [n/24h]	91 (24-256)	15 (3-103)^***^	17 (2-83)^***^	<0.001	90 (2-290)	10 (1-96) *	13 (1-72) *	<0.001	n.s.	91 (53-219)	62 (7-165)^**^	51 (1-114)^***^	<0.001
>76 SVPBs/24h	32 (71%)	17 (38%)^***^	14 (31.1%)^***^	<0.001	13 (65%)	6 (30%)^*^	3 (15%)^*^	<0.001	n.s.	19 (76%)	11 (44%)^*^	10 (40%)^**^	<0.001
VPBs [n/24h]	8 (1-125)	2 (0-27)^***^	1 (0-6)^***^	<0.001	3 (0-55)	1 (0-17)	1 (0-4) *	0.009	n.s.	14 (2-155)	4 (0-74)^*^	0 (0-47)^***^	<0.001
<3 VPBs/24h	18 (40%)	23 (51.1%)	31 (68.9%)^* #^	<0.001	10 (50%)	12 (60%)	14 (70%)	0.05	n.s.	8 (32%)	11 (44%)	17 (68%)^*^	<0.001

Data are presented as mean ± SD, median (IQR) or n(%). *, **, and *** denote respective significance levels of <0.05, <0.01, and <0.001 for comparisons with pre-PTX values; # significantly different versus one-month post-PTX, p<0.05; $ p for the comparison in pre-PTX values between patients with and without HT; bpm, beats per minute.

**Figure 1:**
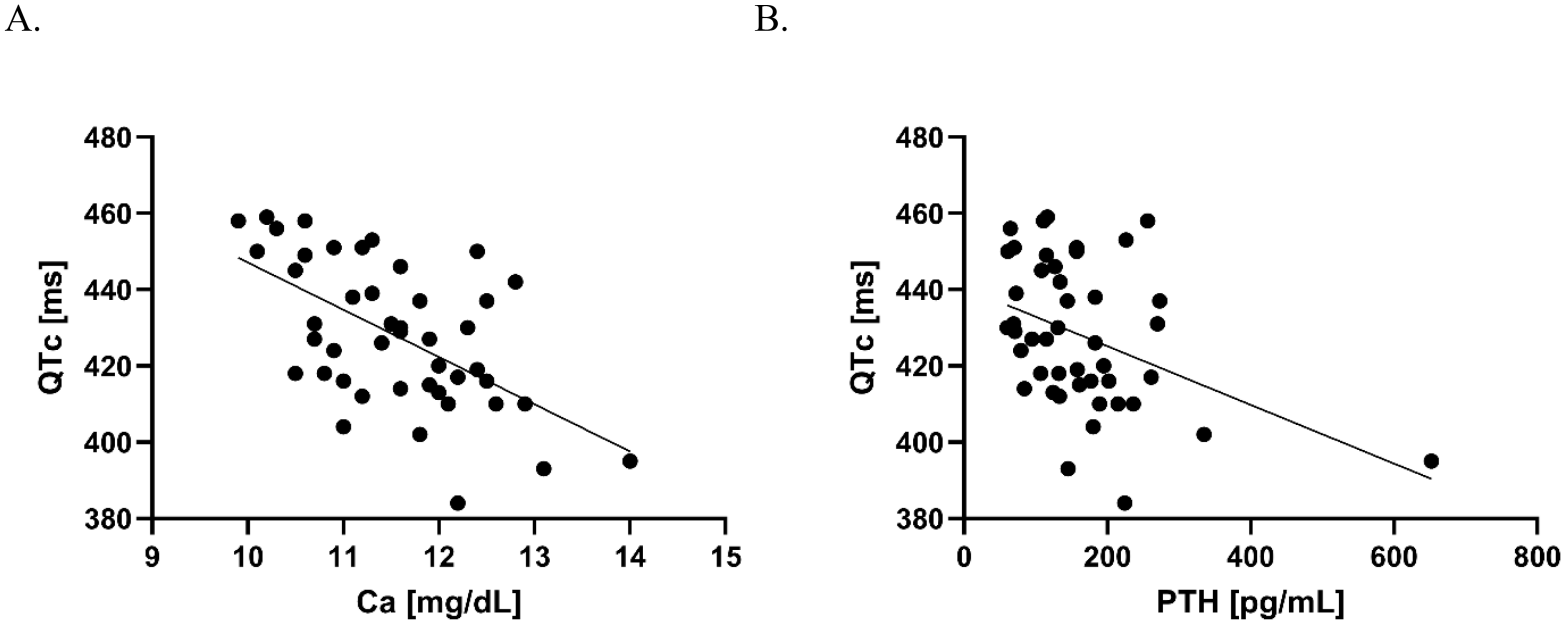
Correlation between Ca and QTc **(A)**, and PTH and QTc **(B)** before PTX. **(A)** Pearson’s correlation coefficient R= –0.58, p<0.001, the line fitted based on simple linear regression: Y = –12.39*X + 571.0; **(B)** Spearman’s correlation coefficient r = –0.28, p<0.01, the fitted line based on simple linear regression: Y = –0.07697*X + 440.6.

Elevated baseline Ca and PTH concentrations decreased significantly after PTX (**Table 1**). Before surgery, Ca and PTH correlated negatively with QTc as demonstrated in **Figure 1**. There were no other correlations between laboratory and Holter ECG parameters.

At all three examination timepoints, sinus rhythm was present in 43 subjects, AF in one, and stimulated rhythm in another. Neither pauses above 2 s nor ST segment abnormalities were registered in any patient. Mean basal HR was 75.4 ± 7.5 bpm. Both median SVPBs and VPBs decreased significantly after PTX. The prevalence of clinically significant SVPBs per 24h among the cohort was 71% before and approximately 34% after PTX, p<0.001, whereas the number of patients with <3VPBs/24h increased from 40% before to 51% one month and 69% six months after surgery, p=0.05. There were no patients with 3 VPBs in 24-h monitoring before and six months after PTX, which allowed for a clear separation of patients based on this number. QTc increased from 428 ± 19 before to 441 ± 17 ms after PTX (p<0.001). QRS complex duration did not change after PTX.

Comparisons between 20 pHPT patients with normotension and 25 pHPT patients with HT revealed that the former had: lower BMI: 23.2 (20.3-25.4) versus 26.7 (24.8-28.4), p=0.001, higher total calcium: 11.9 ± 0.8 versus 11.3 ± 0.9 mg/dL, p<0.001, and shorter QTc: 418 ± 17 versus 436 ± 17 ms, p<0.001.

Surgical pHPT cure lead to an increase in QTc in normotensive patients, whereas no change was recorded in those with HT. An opposite finding was stated for HR (decrease in patients with NT post-PTX, no statistically significant change in patients with HT).

The change in the median number of SVPBs and VPBs was evident both in patients with and without HT. Improvement was comparable between these groups six months post-PTX with respect to the prevalence of those with a clinically significant number of SVPBs (decrease from 65% to 15% in patients without HT versus from 76% to 40% in patients with HT, p=0.31) as well as in the prevalence of patients with fewer than 3 VPBs per 24h (increase from 50% to 70% in patients without HT versus increase from 32% to 68% in patients with HT before and after surgery, p=0.27).

## Discussion

The present study demonstrates improvements in the electrical activity of the heart following surgical cure of pHPT, as measured by 24-hour ECG monitoring. The data reveal substantial decreases in both SVPBs and VPBs as well as normalization of QTc after PTX. These results align with previous research.

In particular, Pepe et al. found that six months after PTX, patients (n=13) experienced a significant reduction in both SVPBs and VPBs. This was not observed in patients who were treated conservatively (same n count) (10). All surgically-treated patients had SVPBs at baseline, while only seven had them after PTX. Similarly, nine had VPBs at baseline, but only three had them at follow-up. In a different approach than 24-h ECG monitoring, significant reductions in exercise-induced VPBs were demonstrated post-PTX, which highlights the improvement in electrophysiologic activity (5, 11).

In our cohort, PTH and Ca correlated negatively with QTc before PTX. The latter correlation was recorded in multiple studies (e.g. Pepe et al. and Lind et al.) (5, 10, 12). Also, the post-PTX QTc prolongation (normalization) observed in the current study is in line with previous reports (5, 13–15).

In studies on ECG abnormalities in the course of pHPT by other authors, normotensive and hypertensive patients have not been considered separately. Here, this approach was applied since we previously demonstrated differences between these groups in systolic and diastolic LV function as well as blood pressure following PTX (8). Certainly, the presence of HT (and associated LV hypertrophy) does affect the risk of arrhythmia (16). However, in our study, pHPT patients with HT also had higher BMI and at the same time lower calcemia compared to those with NT. These differences complicate interpretation of comparable improvement in the rate of clinically significant SVPBs and prevalence of VPBs. Shorter QTc in patients with NT is probably attributable to higher calcemia compared to patients with HT: in a recent study QTc was longer in normocalcemic compared to hypercalcemic pHPT patients (17). Baseline differences between the two subgroups (with NT and HT) of our cohort render drawing generalizable conclusions difficult.

Several other limitations of the present study should be acknowledged. First, the cohort was considerably heterogeneous in terms of pHPT severity, age, and BMI. Second, no controls were included (neither conservatively treated pHPT patients, nor individuals without this endocrinopathy). Third, the follow-up period of six months, while informative, does not capture long-term CV outcomes. The adopted study inclusion criteria excluded patients with normocalcemic pHPT. Including such patients in future analysis would increase the validity of the study, as they represent a clinically important subpopulation of pHPT.

## Conclusions

In conclusion, our findings align with previous research that demonstrate that successful surgical cure of pHPT leads to improvements in cardiac electrical activity, including reductions in arrhythmic burden and normalization of QTc intervals. Bearing in mind concomitant significant improvements in LV function and HT control we reported previously, expanding the indications for PTX may be beneficial (8). Accumulated evidence associates pHPT with CV disorders and a substantial body of research – though not unequivocal – indicates significant CV risk reduction following PTX (1, 16, 18–20). Future studies with larger cohorts and extended follow-up are needed to confirm the beneficial effects of surgical cure of pHPT on the CV system.

## Data Availability

The original contributions presented in the study are included in the article/supplementary material. Further inquiries can be directed to the corresponding author.
